# Cellular indexing of transcriptomes and epitopes (CITE-Seq) in hidradenitis suppurativa identifies dysregulated cell types in peripheral blood and facilitates diagnosis via machine learning

**DOI:** 10.21203/rs.3.rs-4791069/v1

**Published:** 2024-09-09

**Authors:** Sugandh Kumar, Faye Orcales, Bobby B. Shih, Xiaohui Fang, Congcong Yin, Ashley Yates, Peter Dimitrion, Isaac Neuhaus, Chandler Johnson, Indra Adrianto, Antonia Wiala, Iltefat Hamzavi, Li Zhou, Haley Naik, Christian Posch, Qing-Sheng Mi, Wilson Liao

**Affiliations:** 1 Department of Dermatology, University of California at San Francisco, San Francisco, CA, USA; 2 Center for Cutaneous Biology and Immunology, Department of Dermatology, Henry Ford Health, Detroit, MI, USA.; 3 School of Medicine, Wayne State University, Detroit, MI, USA.; 4 Center for Bioinformatics, Department of Public Health Sciences, Henry Ford Health, Detroit, MI, USA.; 5 Immunology Research Program, Henry Ford Cancer Institute, Henry Ford Health, Detroit, MI, USA.; 6 Department of Medicine, College of Human Medicine, Michigan State University, East Lansing, MI, USA.; 7 Henry Ford Health + Michigan State University Health Sciences, Detroit, MI, USA.; 8 Clinic Landstrasse, Department of Dermatology, 1030 Vienna, Austria; 9 School of Medicine, Sigmund Freud University, 1020 Vienna, Austria

**Keywords:** hidradenitis suppurativa, single cell, CITE-seq, machine learning, diagnostic test, pathway enrichment analyses, cell-cell interaction

## Abstract

Hidradenitis suppurativa (HS) is a chronic inflammatory skin condition characterized by painful nodules, abscesses, and scarring, predominantly affecting intertriginous regions and it is often underdiagnosed. This study aimed to utilize single cell RNA and cell-surface protein sequencing (CITE-Seq) to delineate the immune composition of circulating cells in Hidradenitis suppurativa (HS) peripheral blood compared to healthy controls. CITE-Seq was used to analyze the gene and protein expression profiles of peripheral blood mononuclear cells (PBMCs) from 9 HS and 29 healthy controls. The study identified significant differences cell composition between HS patients and healthy controls, including increased proportions of CD14+ and CD16+ monocytes, cDC2, plasmablasts, and proliferating CD4+ T cells in HS patients. Differential expression analysis revealed upregulation of inflammatory markers such as TNF, *IL1B*, and *NF-κB* in monocytes, as well as chemokines and cell adhesion molecules involved in immune cell recruitment and tissue infiltration. Pathway enrichment analysis highlighted the involvement of IL-17, IL-26 and TNF signaling pathways in HS pathogenesis. Machine learning identified key markers for diagnostics and therapeutic development. The findings also support the potential for machine learning models to aid in the diagnosis of HS based on immune cell markers. These insights may inform future therapeutic strategies targeting specific immune pathways in HS.

## 1 Introduction

Hidradenitis suppurativa (HS) is a chronic inflammatory skin condition characterized by painful lesions, including nodules, abscesses, skin tunnels, and scarring^1^. These manifestations primarily affect intertriginous areas, notably the axillary, groin, perianal, perineal, and inframammary regions. The impact of the condition extends beyond physical discomfort; the associated pain, drainage, malodor, and scarring often lead to profound negative psychosocial effects. The estimated global prevalence of HS is 1–2%, but it is likely underreported due to underdiagnosis and inadequate treatment. HS is also associated with comorbidities including psoriasis, Crohn’s disease, metabolic syndrome, and spondyloarthritis^2^.

Single-cell technologies have generated large immunological datasets to delineate the biological mechanisms of immune-mediated disease pathways at high resolution.^3 4^. In HS, a study by Straalen et al. identified *TNF-α*, *IL-1β*, *SFRP4*+, and *CXCL13*+ as relevant therapeutic targets for HS patients through spatial sequencing and complement analysis^5^. Another study explored inflammatory responses in HS, revealing that the immune responses were centered on *IFN-γ, IL-36,* and TNF, with a lesser contribution from *IL-17A*^6^.

Treatment options for HS remain limited. Currently, two drugs, adalimumab and secukinumab, are the only FDA approved targeted therapies for HS. Briefly, adalimumab acts as a TNF inhibitor, achieving a clinical response in just 40 to 60% of patients. Secukinumab, an inhibitor targeting the *IL-17A*, plays a significant role in the pathophysiology of HS by preventing interaction with the *IL-17* receptor ^7^.

In this study, we utilized single-cell cellular indexing of transcriptomes and protein epitopes (CITE-Seq) to define the immune composition of circulating cells in HS peripheral blood compared to healthy controls. Our results provide a high-resolution view of HS systemic immunity, identifying dysregulated cell types and novel cell-cell interactions that contribute to disease inflammation. We also identify HS pathways that may serve as potential targets for future therapeutic intervention. Finally, we developed several machine learning models for the accurate classification of HS and healthy individuals based on the expression of multiple cell markers, thereby advancing biomarker discovery.

## 2 Methodology

### 2.1 Subject Recruitment and Sampling

Peripheral blood mononuclear cells (PBMCs) were collected from nine HS subjects at Henry Ford Hospital, Detroit, Michigan, USA. The HS subjects comprised 6 females and 3 males with mean age 43.0 (SD 13.2). All subjects were either Hurley Stage II or III. All subjects provided written informed consent (Henry Ford Health IRB# 12826). The clinical diagnosis of HS was confirmed by a dermatologist. None of the subjects were receiving biologic or systemic therapy for HS during PBMC collection. Additionally, PBMCs from 29 healthy controls, age- and sex-matched, were collected at the University of California San Francisco under IRB approval 10–02830. Detailed subject information is in Supplementary Table 1. Peripheral blood was collected in Vacutainer ACD tubes, PBMCs were isolated using Ficoll, and stored in liquid nitrogen.

### 2.2 Sample and Library Preparation for CITE-Seq Sequencing

For each sample, 500 μL of thawed PBMCs were mixed with 10 mL of EasySep buffer (StemCell Technologies, Cat. 20144) and centrifuged at 300g for 5 minutes at room temperature. The resulting cell pellets were resuspended in 1 mL of a buffer solution containing 18 mL EasySep and 21 μL Benzonase Nuclease (MilliporeSigma, Cat. 70664) to break down extracellular nucleic acids. This mixture was incubated at room temperature for 15 minutes. The cell suspensions treated with nuclease were then passed through a 40 μm Flowmi Cell Strainer (Bel-Art, Cat. H13680–0040), centrifuged again at 300g for 5 minutes at room temperature, and resuspended in 100 μL EasySep buffer. Final cell counts were obtained by diluting the suspensions 1:100 and staining with 0.4% trypan blue, followed by counting with a Countess I FL Automated Cell Counter (Thermo Fisher Scientific).

cDNA libraries for gene expression were prepared following the manufacturer’s guidelines (Chromium Next GEM Single Cell 3’ v3.1), including 12 cycles of PCR amplification. Cellometer (Nexcelom - Lawrence, MA) was used to determine the cell viability and concentration to normalize to 1E6 cells/ml, 40,000 cells were targeted for each library. For antibody-derived tags (ADT) libraries from feature barcoding antibodies, size purification was repeated on the supernatant from the initial cDNA library size purification, using a 7:8 volumetric ratio of 2.0X SPRIselect reagent (Beckman Coulter, Cat. B23317) to the sample. Indexing amplification was carried out using Kapa HiFi HotStart ReadyMix (Kapa Biosystems, Cat. KK2601) and TruSeq Small RNA RPI primers (Illumina) under the following conditions: initial denaturation at 98°C for 2 minutes, followed by 15 cycles of 98°C for 20 seconds, 60°C for 30 seconds, and 72°C for 20 seconds, and a final extension at 72°C for 5 minutes. The amplified libraries underwent another round of size purification using a 5:6 volumetric ratio of 1.2X SPRIselect reagent to the sample. Libraries were quantified with a Bioanalyzer 2100 (Agilent) and sequenced on a NovaSeq 6000 (Illumina).

### 2.3 Antibody-Tag protein Sequencing

For the antibody-tagged protein 10X sequencing, staining of cell surface protein was performed using a modified version of the Totalseq-A protocol (details at https://www.biolegend.com/en-us/protocols/totalseq-a-dual-index-protocol). A pooled cell suspension, comprising 100,000 cells from up to 10 subjects, was centrifuged at 300g for 5 minutes at 4°C. The cell pellets were then resuspended in 100 μL Cell Staining Buffer (BioLegend, Cat. 420201) and incubated for 10 minutes at 4°C with 10 μL Human TruStain FcX^™^ Fc Blocking Solution (BioLegend, Cat. 422301). Following this, the cell suspensions were stained for 30 minutes at 4°C with 100 μL of a TotalSeq antibody cocktail. The stained cells were then divided into two aliquots of 105 μL each.

Each aliquot was subjected to three washes by resuspending the cells in 15 mL of Cell Staining Buffer and centrifuging at 300g for 5 minutes at 4°C. After washing, the cells were resuspended in 150 μL of 10% FBS in PBS, recombined, and filtered through a 40 μm Flowmi Cell Strainer. To assess cell viability, 10 μL of the filtered cells were mixed with 10 μL of 0.4% Trypan Blue and counted manually using a hemocytometer. The cell density was then adjusted to 2,500 cells/μL, and the cells were processed on the Chromium Controller (10X Genomics) using the Single Cell 3’ v3.1 Assay (10X Genomics), targeting 50,000 cells per reaction.

### 2.4 Genotyping of PBMCs

Genomic DNA was extracted from peripheral blood mononuclear cells (PBMCs) using the DNeasy Blood and Tissue Kit (Qiagen, Cat. 69504) according to the manufacturer’s instructions. The concentration and purity of extracted DNA quality were determined using a NanoDrop 2000 spectrophotometer (Thermo Fisher Scientific). Further, the extracted DNA samples were genotyped using the Affymetrix UK Biobank Axiom Array (ThermoFisher Scientific) on a GeneTitan Multi-Channel Instrument (Applied Biosystems) following the recommended protocols.

### 2.5 Genotype Calling and Quality Control

The genotype samples from the DNA array were processed using Thermo-Analysis Power Tools 2.10.2.2 (Affymetrix) to generate genotype calls. Standard quality control filters were applied, including sample call rate >98%, SNP call rate >98%, minor allele frequency >1%, and Hardy-Weinberg Equilibrium p-value > 1×10^6. The resulting genotype data files (VCFs) were further scanned using snpflip (https://github.com/biocore-ntnu/snpflip) against the GRCh37 human genome reference sequence from the University of California, Santa Cruz (http://hgdownload.cse.ucsc.edu/goldenPath/hg19/bigZips/hg19.fa.gz). Reversed and ambiguous-stranded SNPs were identified, flipped, and removed, respectively, using Plink 1.90 (http://pngu.mgh.harvard.edu/purcell/plink/). The remaining SNPs were sorted using Plink 2.00a3LM (www.cog-genomics.org/plink/2.0/). Additional untyped SNPs were imputed using the Michigan Imputation Server (https://imputationserver.sph.umich.edu) with the 1000G Phase 3 v5 GRCh37 reference panel, Eagle v2.4 phasing, and the EUR population. The SNP positions were then converted to GRCh38 coordinates using the ‘LiftoverVcf’ command of Picard 2.23.3 (http://broadinstitute.github.io/picard/). Finally, Vcftools 0.1.13 was used to exclude non-exonic SNPs and SNPs with minor allele frequency < 0.05. The resulting high-quality genotype data were used for demuxlet of sequencing data.

### 2.6 Cell Demultiplexing and Doublet Removal

We sequenced multiple samples into a single aliquot per Chromium run, resulting in multiple samples combined into one count matrix. To determine the subject of origin for all droplet barcodes within each RNA count matrix, we used ‘demuxlet’ as part of the ‘popscle’ suite (https://github.com/statgen/popscle). This R-package, applied to imputation-augmented exonic SNP genotypes as described earlier, enabled the exclusion of doublets detected between different individuals. The count matrices for each Chromium library were then imported into R for further analysis using the ‘Seurat’ 5.0.0 R package. The demuxlet identified heterogenic doublets, which are doublets formed from cells originating from different donors^8^. Additionally, the ‘Doubletfinder’ V2.0 into R package was utilized to remove doublets formed by both heterogenic and homogenic doublets, as it relies on transcriptional signatures rather than genetic variation.

### 2.7 Clustering and Annotation of Clusters

ADT expression was estimated for cells with measured RNA but not ADT according to the Seurat reference mapping protocol (https://satijalab.org/seurat/articles/multimodal_reference_mapping.html), and unless otherwise noted, all function names described here belong to the Seurat package. Briefly, the integrated dataset above was split into the subset of cells with ADT measurements (reference subset) and the subset of cells without ADT measurements (query subset). RNA expression normalization and scaling were performed using ‘SCTransform’ on both subsets, adjusting for the number of features and total counts in each cell. While ADT expression normalization for the reference subset was performed using the Centered Log-Ratio (CLR), followed by mean centering and scaling. For the reference subset, PCA was then run for both the SCTransformed RNA (SCT) expression and the ADT expression, and a weighted nearest-neighbor network for the reference subset was calculated from first 30 for and ADT, respectively, using the ‘FindMultiModalNeighbors’ function. Next, SCT from the reference subset was transformed again using supervised PCA (via the ‘RunPCA’ function) to identify the principal components that best capture the combined RNA and ADT expression variation represented by the weighted nearest-neighbor network.

### 2.8 Cluster Annotation through Azimuth

After QC, all samples were merged into one object and cluster annotation was performed through Azimuth using labels from the Human PBMC reference^9^.

### 2.9 Samples Batch-correction and Integration

Batch effects were corrected via integration of SCT expression data according to the Seurat integration protocol (https://satijalab.org/seurat/articles/seurat5_integration). Briefly, samples were split, and 3000 genes consistently variable among the individual SCT matrices were selected using SelectIntegrationFeatures. PrepSCTIntegration was then used to prepare reduced SCT expression matrices for these genes. PCA was calculated for each reduced SCT matrix using RunPCA, and the first 30 principal components were used to identify transcriptionally similar cells between each pair of reduced SCT matrices. The *IntegrateLayers* function and *Harmony* method were then used to integrate the SCT matrices, and UMAP was re-generated to visualize the batch effect between HS and Healthy samples.

### 2.10 Cell Cluster Proportion Comparison

We compared the cell type proportions of each cluster within each subject group relative to their total cell count. To determine significant differences in cell proportions among the subject cohorts, we employed the Kruskal-Walli’s test (*kruskal.test in R*). Each cell types with FDR-adjusted *Kruskal-Wallis p-values* less than 0.05 were considered as statistically different between the cohorts group.

### 2.11 Differential Feature Analysis of genes and cell-surface marker

Differentially expressed genes (DEGs) and proteins (DEPs) were identified using the *FindMarkers* function in *Seurat*. Separate analyses for DEGs and DEPs were performed, and each cell type included in the analysis had at least 100 cells. The non-parametric Wilcoxon rank sum test (*test.use* = *“wilcox*”) was used for identifying DEGs and DEPs. Genes were considered DEGs if they had both a Bonferroni-corrected p-value < 0.001 and an absolute log fold change > 1.5. Proteins were considered DEPs if they had a p-value < 0.005 and an absolute log fold change > 1.0.

### 2.12 Biological pathways and gene ontology analysis

Pathway and functional enrichment analysis was performed with the g:Profiler tool g:GOST using gprofiler2 in RStudio (v4.1.0). Functional enrichment analysis was performed separately for significantly up- and down-regulated genes (log2FC > 1.5 & p_value < 0.01) for each individual cell type with at least 100 cells. Input gene lists were ranked by log2FC and g:GOST was performed as an ordered query using KEGG, Reactome, and WikiPathway terms. Terms were then filtered for significance, retaining terms with query_size > 50, term_size > 10, and p_value < 0.05. Precision was defined as *intersection_size / query_size* and recall was defined as *intersection_size / term_size*, where *query_size* refers to the n number of top genes (determined by ranking) that are evaluated by g:GOST for a given term, *intersection_size* refers to the number of genes within *query_size* that match a given term, and *term_size* refers to the number of genes contained within a term.

### 2.13 Cell-Cell Interaction analysis

Cell-cell interactions were inferred using CellPhoneDB (v5.0.0) ^10,11^ and querying a manually curated repository of receptors, ligands, and their interactions (cellphonedb-data v5.0.0). CellPhoneDB was performed separately for HS and healthy cells, providing log-normalized counts and Azimuth cell type annotations. CellPhoneDB analysis was run using the statistical_analysis method with default settings. Briefly, CellPhoneDB performs 1000 random permutations of cell labels to estimate a null distribution of the mean of the average ligand and receptor expression (*mean_exp*) in the interacting clusters and estimate a p-value. A given ligand and receptor pair are only considered if both are expressed in at least 10% of the corresponding cell type. We filtered for significant HS enriched interactions by only considering cell-cell interactions in which *mean_exp* is at least 10% greater in HS as compared to healthy and with a p-value of less than 0.05 in HS. Here, we only considered cell-cell interactions in which both cell pairs have at least 10 cells in both HS and healthy.

### 2.14 Machine learning classification model

Machine learning (ML) models were used to classify HS vs healthy patients, including Random Forest Classifier (RF), Gradient Boosting Classifier (GB), Support Vector Machine for Classification (SVC), and Neural Network (NN). *sci-kit learn (v1.4.2)* was used for all classifier models, except the NN model which was from the *keras (v3.3.3)* library in *python3.4*. The data were split into 60:40, as a Training and Test set. Data inputted into the ML models included differentially expressed genes and proteins, with their means normalized and centered, and expression values scaled. These expression values were calculated for each patient across all present cell types.

Each model underwent training and testing with a set of RNA expression only data, and was then repeated for an ADT only dataset, and finally a combined RNA + ADT expression dataset. Only training data were used to build the ML models while the test data were used for independent evaluation of prediction models. A five-fold cross validation step with hyperparameter tuning was performed on each model. Hyperparameters tested for RF included *n_estimators* = (100, 200, 500, 1000), GB included *learning_rate* = (0.1, 0.2, 0.4, 0.8), SVC included kernel = (*linear, poly, rbf, sigmoid*), and NN included *learning_rate* = (0.001, 0.0001) and *rate* = (0.4, 0.8). The evaluation metric used to determine the best performing models was accuracy. The best performing models were then used to extract the top 20 markers for each dataset (RNA only, ADT only, and RNA + ADT). An AUC-ROC curve was plotted for only the top performing models to calculate classification rate.

## 3 Results

### 3.1 Single-cell transcriptomic analysis of 29 cell types in HS vs healthy blood identifies cell composition differences in monocytes, T cells, B cells, dendritic cells, NK cells, and innate lymphoid cells

**Figure 1.**
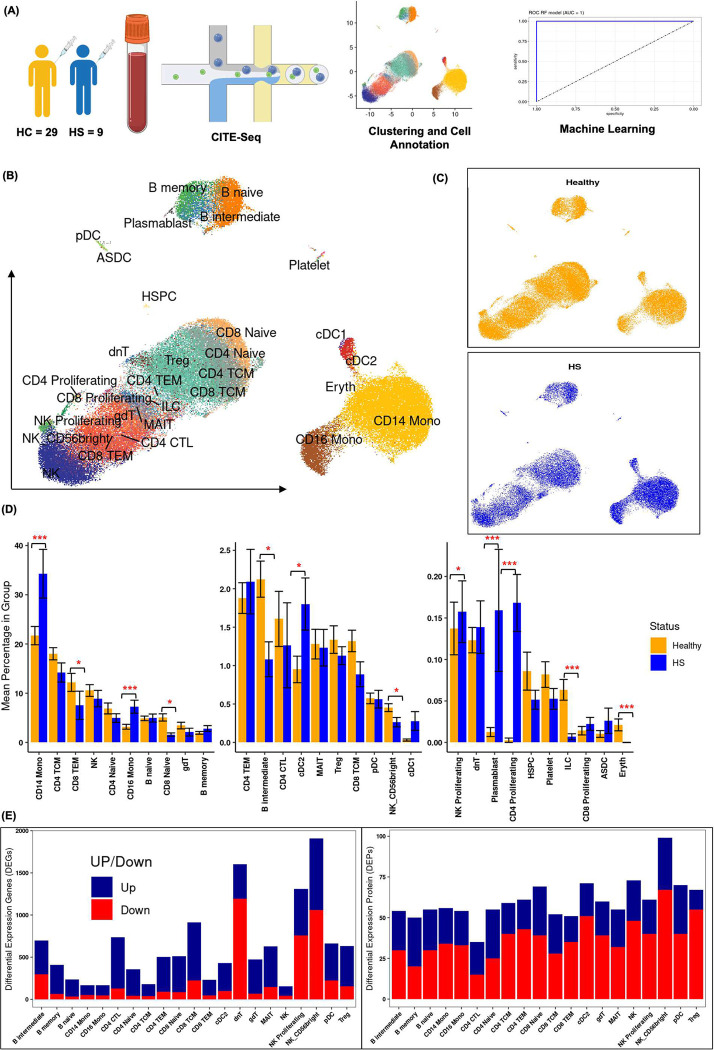
**(A)** The study design is outlined, showing the process from patient recruitment to sample analysis. Peripheral blood mononuclear cells (PBMCs) were collected from 9 HS patients and healthy controls for comparison. **(B)** Thirty different cell types were annotated using Azimuth and visualized through UMAP based on SCTransform-normalized RNA expression integrated with ADT expression, with distinct colors representing different cell subsets. **(C)** UMAP plots depict the clustering of cell types in HS and healthy samples, highlighting differences between the two groups. **(D)** The mean percentages of each cell type within the total PBMCs of each subject are shown, with error bars indicating the standard error of the mean. Significant differences between HS and healthy subjects were determined using Wilcoxon and FDR-adjusted Kruskal-Wallis tests, with * indicating p-values < 0.05. **(E)** The number of differentially expressed genes (DEGs) and proteins (DEPs) in HS vs. healthy samples across all cell types are presented, with separate counts for upregulated (blue) and downregulated (red) genes and proteins.

To delineate the different immunological features from 9 HS patients and 29 healthy controls, droplet-based single-cell RNA-sequencing (10X Genomics) was conducted to examine the gene expression patterns. The workflow is depicted in [Figure 1A]. After quality control (see Methods), 109,873 high-quality single cells with distinct transcriptome profiles were obtained, with 34,545 cells from 9 HS subjects and 75,303 cells from 29 healthy controls. Batch effected correction was done at the sample as well as subject wise [Supplementary Figure 1–2]. After quality control on the cell-surface antibody-tagged protein data, 258 high-quality protein markers were retained for both sample groups. As shown in [Figure 1B], 29 major PBMC cell types were delineated based on the expression of canonical gene markers in Azimuth. Both HS and healthy subjects harbored all 29 cell populations including monocytes (CD14+ and CD16+), CD4+ T cells (naive, central memory, effector memory, cytotoxic, and proliferating), CD8+ T cells (naive, effector memory, and central memory), regulatory T cells, gamma-delta T cells, double-negative T cells, mucosal-associated invariant T cells (MAIT), natural killer cells (NK, NK CD56bright, and proliferating NK), B cells (memory, naive, and intermediate), conventional dendritic cells (cDC1 and cDC2), plasmacytoid dendritic cells, plasmablasts, hematopoietic stem and progenitor cells, platelets, innate lymphoid cells, and erythrocytes. The details of cell cluster composition [Supplementary Figure 3] in HS and healthy and top three marker genes for each cell cluster are shown in dot expression plot [Supplementary Figure 4].

We calculated the mean percentage composition in each of the 29 clusters in HS and compared the percentages to those of healthy control samples. The analysis revealed significant differences in the abundance of several immune cell populations between HS and healthy individuals [Figure 1C]. In comparison to healthy controls, HS subjects had a significantly higher proportion of CD14+ monocytes, CD16+ monocytes, cDC2, cDC1, plasmablasts, and proliferating CD4+ T cells. In contrast, HS subjects had a significantly lower proportion of CD8+ effector memory T cells (CD8 TEM), CD8+ naive T cells, B intermediate cells, CD56bright NK cells, and innate lymphoid cells (ILC). These findings indicate that various immune cell subsets, including monocytes, T cells, B cells, dendritic cells, NK cells, and innate lymphoid cells, exhibit differential abundance in the peripheral blood of HS patients compared to healthy individuals [Figure 1 D].

### 3.2 Differential expression of RNA and protein reveals altered immunity in HS

Differential expression analysis of genes and proteins was performed on all 29 cell types and the list of DEG and DEP are in (Supplementary Table 2 and 3). However, we focused our analysis on cell types with differential cell abundance and cell types with at least 100 cells per cluster (CD14+ monocytes, CD16+ monocytes, CD8+ TEM, CD8+ T Naïve, B Intermediate, cDC2, NK Proliferating, and NK 56 bright). The numbers of differentially expressed genes and protein of these cell types are shown in [Figure 1E].

**Figure 2.**
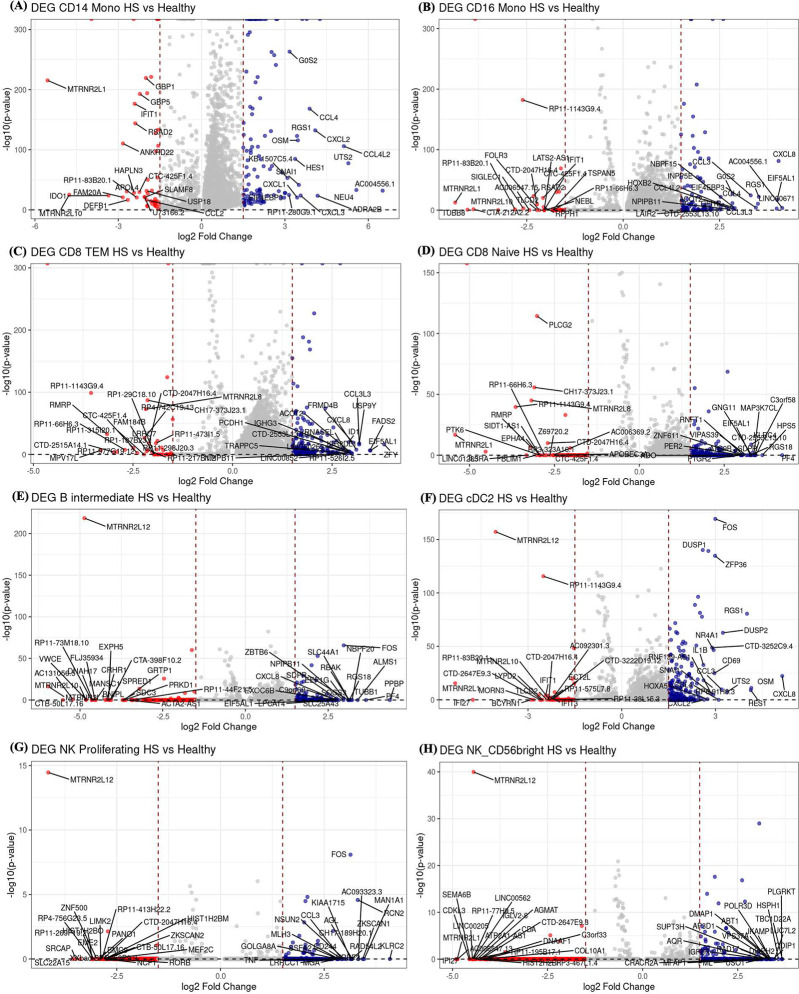
Volcano plot of differentially expressed genes in various cell types. The volcano plots of differentially expressed genes in HS versus healthy controls across several cell types: (A) CD14 monocytes, (B) CD16 monocytes, (C) CD8 TEM cells, (D) CD8 naïve cells, (E) B-intermediate cells, (F) cDC2 cells, (G) NK proliferating cells, and (H) NK56 bright cells. In eachplot, blue dots represent upregulated genes in HS, while red dots indicate downregulated genes. The top 20 genes for both upregulation and downregulation are labeled based on their p-values.

Within CD14+ monocytes, there was overexpression of the inflammatory markers ***TNF*, *IL1B***, and ***NF-κB***; chemokines ***CCL2*, *CXCL8***, and ***CCL5***; cell proliferation and migration markers ***MKI67, MMP9***, and ***CDK1***; and cell adhesion molecules ***ITGB2*, *ICAM1***, and ***VCAM1***. Similarly, CD16+ monocytes showed upregulation of ***TNF*, *ZFP36*,** and ***IL1B***; ***DUSP1***, a phosphatase regulating **MAPKs**; and the chemokines and cytokine ***CCL4*, *CXCL8*,** and ***IL6***. The cell adhesion genes ***MKI67, VCAM1, ITGA4***, and ***CD44*** were upregulated, as were metabolic genes ***LDHA, GAPDH***, and ***PKM***. ***NOD2***, relevant to HS pathology, was also upregulated [Figure 2 A–B].

**Figure 3.**
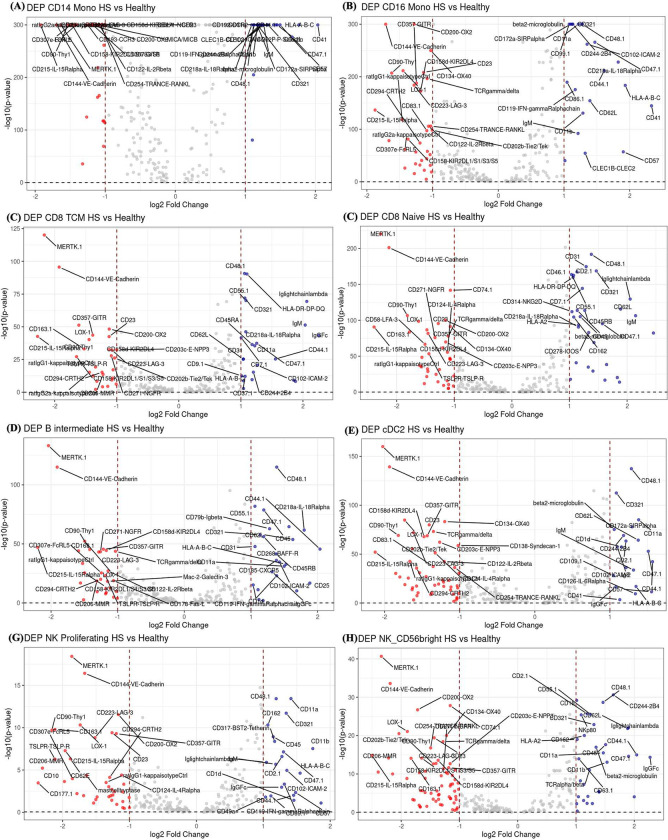
Volcano plot of differentially expressed proteins (DEPs) in various cell types. The volcano plots of differentially expressed proteins (DEPs) in HS versus healthy controls across several cell types: (A) CD14 monocytes, (B) CD16 monocytes, (C) CD8 TEM cells, (D) CD8 naïve cells, (E) B-intermediate cells, (F) cDC2 cells, (G) NK proliferating cells, and (H) NK56 bright cells. In each plot, blue dots represent upregulated proteins in HS, while red dots indicate downregulated genes. The top 20 genes for both upregulation and downregulation are labeled based on their p-values.

Within CD8+ Naive and CD8+ TEM cells, there was upregulation of ***FKBIA*** and ***CCL4L2***, involved in cell recruitment, and ***CD69*** [Figure 3C–D]. Proliferation and migration genes ***PMAIP1, PPP4C***, and ***PHLDA1*** were upregulated, as was ***RHOB***. DEGs in B-intermediate cells included ***NF-κB***, a key inflammatory pathway regulator; ***JUNB***, influencing cytokine production; and ***FOS***, part of the AP-1 complex. There was also upregulation of ***CD69, DUSP1, DUSP2*,** and ***AREG*** involved in cell recruitment and migration. ***S100A9***, a calcium-binding protein that promotes T17 responses, and ***TNFSF12***, a TNF ligand, were upregulated. [Figure 2 E–F]

Within cDC2 cells, there was upregulation of the inflammatory chemokines ***CXCL8*, *CCL3*, *CCL4*** and cytokines ***IL1B, IL18RAP***. NK cells had significant upregulation of the inflammatory mediators ***NF-κB****,* ***FOS***, and ***IL6*;** cytotoxicity genes ***GZMB, PRF1***, and ***CD69***; and adhesion and migration genes ***ITGA4, ITGB2*,** and ***ICAM1*** [Figure 2 F].

Differential expressed proteins (DEPs) in CD14+ and CD16+ monocytes included integrins such as **CD41, CD11a**, and **CD11b**. The adhesion molecules **CD62L**, **CD44.1**, and **ICAM-2** were also elevated in many cell types, suggesting enhanced interactions with endothelial cells and tissue infiltration. Several cell surface markers were also found downregulated including **CD83.1, CD137L-4–1BBLigand, CD177.1**, and **CD134-OX40** expressed in monocytes, CD8 Naïve and TEM, NK and Treg, which play critical roles in immune cell activation and regulation. Additional downregulated proteins were **CD202b-Tie2/Tek, CD254-TRANCE-RANKL**, and **CD223-LAG-3** [Figure 3A–H].

### 3.3 Pathway enrichment analysis identifies pro-inflammatory signatures and points to a significant role of CD14 and CD16 monocytes

**Figure 4.**
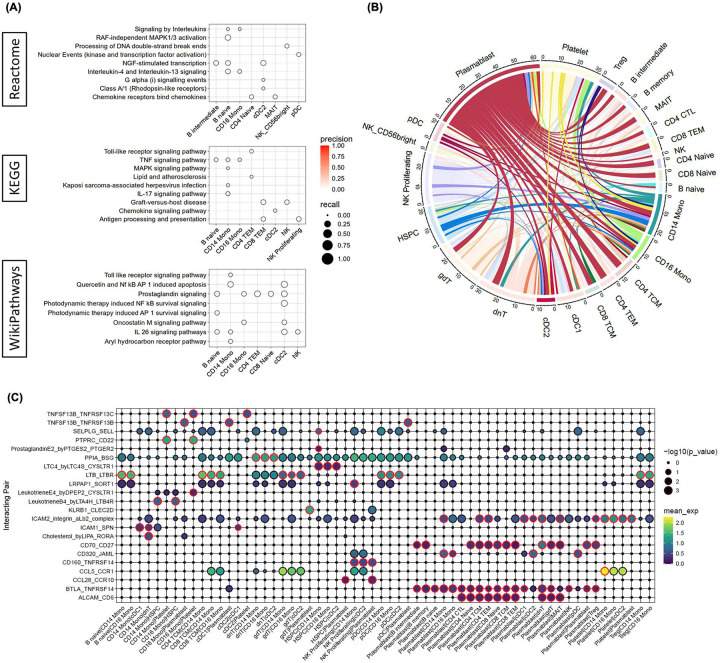
Pathway enrichment analysis for differentially expressed genes (DEGs) in various cell types in HS versus healthy controls. The dot plot shows the precision and recall values for each pathway in (A) Wiki Pathway, REACTOME, andKEGG databases. Key pathways involved in inflammatory signaling and immune responses are highlighted, such as signaling by TNF, interleukins, MAPK1/3 activation, chemokine receptor binding, and IL-4/IL-13 signaling. Each dot represents a specific cell type, including B intermediate, B naïve, CD16 monocytes, CD4 naïve, CD4 memory, CD8 TEM, CD8 naïve, cDC2, MAIT, NK, NK_Proliferating, and NK_CD56bright cells. (B) Circos plot depicting cell-cell interactions between various immune cell types in HS versus healthy controls. The plot visualizes interactions among different cell populations, including CD8 TEM, CD8 TCM, CD8 naïve, CD4 TCM, CD4 naïve, CD4 CTL, CD4 proliferating, CD14 monocytes, CD16 monocytes, cDC1, cDC2, plasmablasts, Treg, and various B cell subsets. The lines indicate interactions, with color-coded segments representing different cell types. The thickness of the lines corresponds to the interaction strength, highlighting the complexity of the immune network in HS. (C) Dot plot representing the average expression and p-value of a given receptor-ligand pair by cell pair. Of note, represented here are expression values and p-values in HS samples only. Dots with a red border represent a specific receptor-ligand interaction between a given cell-cell pair that is significantly enriched in HS compared to healthy, as defined in methods. For a given receptor_ligand pair detected in a cell pair, cellA_cellB, the receptor and ligand are inferred as expressed in cellA and cellB, respectively.

To further elucidate the cell type specific biological pathways underpinning disease pathogenesis in HS compared to healthy control, we performed functional enrichment analysis using g:Profiler (g:GOSt) ^12^. Briefly, for each cell type we performed separate g:GOSt analyses utilizing significantly up- or down-regulated genes (log2FC > 1.5 & pval < 0.01) as a ordered query. Genes were mapped to the gene sets sourced from KEGG, Reactome and WikiPathways databases. We observed positive enrichment of several interleukin signaling gene sets in the transcriptome of CD14/16 monocytes, B naïve cells, and NK cells. Specifically, we observed upregulation of the *IL-17* signaling pathways in CD14/16 monocytes, the *IL-4/13* and *IL-26* signaling pathway in B naïve and CD14/16 monocytes, and the *IL-26* signaling pathway in B naïve, CD14 monocytes, cDC2, and NK cells [Figure 4A]. Significantly, *IL-17* has been shown to be elevated in the serum of patients with HS and positively correlated with disease severity^13^. Additionally, HS is associated with the *Th1/Th17*-driven inflammatory response that acts through downstream signaling pathways *NF-κB*, CCAAT/enhancer-binding protein (C/EBP) family, and mitogen-activated protein kinase (*MAPK*) ^14,15^. Supporting this, we also observed positive enrichment of the *MAPK* and TNF signaling pathway in CD14 monocytes (Figure 4A). Overall, our results suggest a potentially significant role of monocytes in the pathogenesis of HS acting through the *IL-17* signaling pathway.

### 3.4 Cell-cell interaction analysis supports a pro-inflammatory role for monocytes and B-cells mediated through TNF signaling and T-cell activation

To characterize the intercellular interaction landscape in HS, we performed cell-cell interaction analysis using CellPhoneDB ^10,11^. Briefly, CellPhoneDB was run separately for HS and healthy using cell type annotated log-normalized expression values. We filtered for HS enriched cell-cell interactions if a given receptor-ligand pair had at least a 10% greater mean expression in HS compared to healthy and with p-value < 0.05 in HS. This resulted in a total of 188 HS-enriched significant cell-cell interactions, with the most common cell partner being plasmablasts (61) followed by double negative T-cells (31), platelets (31), NK proliferating (27), γδ T cells (26), and CD14 monocytes (23) (Figure 4B). To narrow our search, we focused on cell-cell interactions involving significantly expanded cell types in HS, namely CD14/16 monocytes, cDC2, and plasmablasts (Figure 4C). In concordance with our pathway enrichment analysis, we identified several cell-cell interactions primarily involving CD14/16 monocytes and plasmablasts that mediate signaling through the TNF signaling pathway (*TNFSF13B-TNFRSF13C, TNFSF13B-TNFRSF13B*) (Figure 4C). *TNFSF13B* (BAFF) interactions with *TNFRSF13C* (BAFF-R) and *TNFRSF13B* (TACI) are known to be critical for B-cell survival and activation through engagement with TRAF adaptors and the activation of *NF-κB* and *MAPK* ^16–19^. Notably, BAFF is primarily produced by macrophages, monocytes and dendritic cells, in agreement with our observations of BAFF interactions between CD16 monocytes/cDC1 cells and plasmablasts ^20,21^. Here, we also observe significant interactions between CD70, expressed by plasmablasts, and CD27, expressed by B-cells and T-cells (Figure 4C). Importantly, CD70 expression on activated antigen presenting cells (dendritic cells and B-cells) is known to bind CD27 on T-cells and promote T-cell activation/differentiation and stimulate production of pro-inflammatory cytokines through *TRAF2/5* mediated activation of the *NF-κB* and JNK pathways ^22–24^. Similarly, our data further suggests T-cell activation in HS is mediated by interaction with plasmablasts through the *ALCAM-*CD6 interaction (Figure 4C). *ALCAM* has been shown to support T-cell migration and activation, promoting transendothelial migration and immune responses ^25,26^. Taken together, and in agreement with our functional enrichment analysis, these results support a potentially pathogenic role of monocytes and B-cells mediated by TNF signaling through BAFF and CD70-CD27 interactions.

### 3.5 Machine learning model for classification of HS and Healthy based on top identified markers

**Figure 5.**
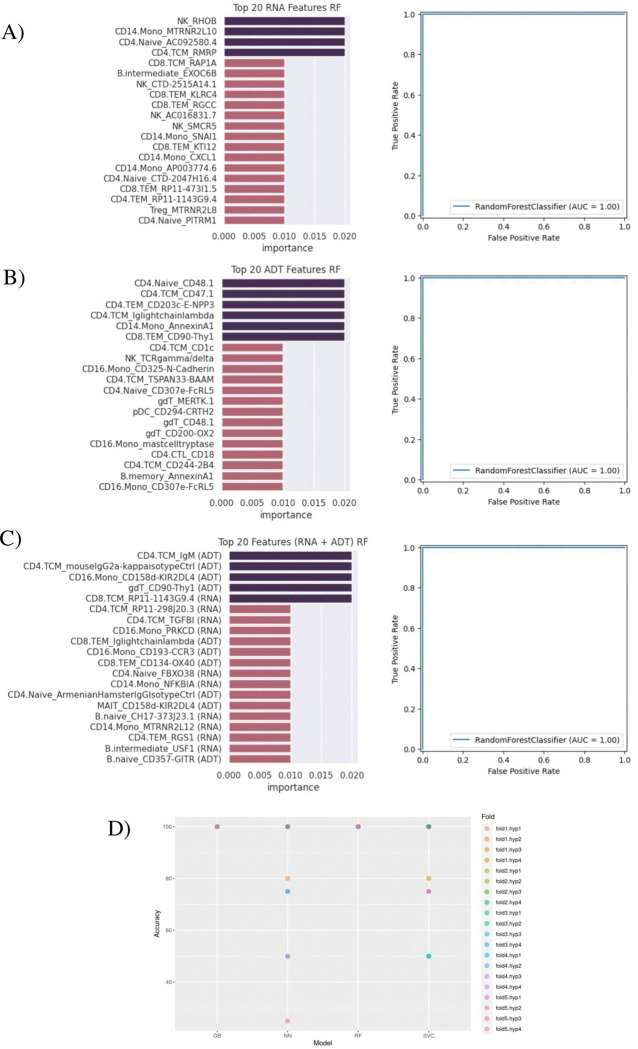
Machine learning models to classify the HS and Healthy Patients based on the identified biomarkers. (A) Top 20 ensemble features predicted using DEGs. (B) Top 20 ensemble features predicted using DEPs. (C) Combined predicted features from DEGs and DEPs. (A-C) Model performance was evaluated using an independent test and accuracy presented in AUC-ROC curves. (D) Accuracies for each cross-validation fold and hyperparameter tuning set are shown for 4 ML models: Gradient Boosting (GB), Neural Network (NN), Random Forest (RF), Support Vector Machine for Classification (SVC).

The top 20 markers for each dataset and each model showed varying results. For RF and GB models, the top 20 markers resulting from the combined gene + protein input data showed a relatively even split between RNA and ADT markers. Both models also had primarily ADT markers as the features with highest importance [Figure 5C, Supplementary Figure 6]. As for the SVC model, it showed only RNA markers populating its top 20 [Supplementary Figure 6].

Using the best performing hyperparameters, all ML models were able to classify with a maximum accuracy of 100% as well as precision, recall, f1, and perfect AUC scores of 1.0. For RF and GB models in particular, accuracies did not go under 100% and AUC scores did not go under 1.0, even after being tested with different hyperparameters and training/validation folds [Figure 5D].

The top RNA discriminators included *RHOB* in NK cells, *MTRNR2L10* in CD14 Monocytes, *PITRM1* in CD4 Naïve cells, and *RMRP* in CD4 TCM cells. The top cell surface protein discriminators included MICA/MICB on CD8 TEM cells, CD314 (NKG2D) on NK cells, AnnexinA1 on CD14 Monocytes, and CD48.1 on CD4 Naïve cells [Figure 5A–B]. The *MTRNR2L12* gene and the CD325 (N-Cadherin) surface protein were top discriminators in multiple different cell types [Supplementary Figure 6].

## 4. Discussion

### 4.1 Characterization of Dysregulated Immune Cell Populations in PBMCs Highlights Key Cell Types

Hidradenitis suppurativa is a highly debilitating and heterogeneous chronic inflammatory disease with diverse clinical presentations and responses to treatment, driven by distinct genetic and transcriptomic mechanisms. While several genetic and functional studies have identified various genetic risk factors and characterized the molecular mechanisms underpinning HS pathogenesis, little is known about the exact cell specific mechanisms that drive chronic and systemic inflammation observed in HS patients ^14,27–30^. To this end, and to the best of our knowledge, this study performs the first single-cell transcriptomic profiling of PBMCs isolated from patients with HS. Our findings underscore the complex interactions among different immune cell types and their regulatory mechanisms, shedding light on their potential roles in the pathogenesis of HS ^5 31 32^.

We identified several immune cells were significantly increased in HS, including CD14 monocytes and CD16 monocytes, cDC1/2, NK proliferating cells, plasmablasts, and CD4 proliferating cells. This suggests the contribution of these cell types to the inflammatory processes and immune dysregulation observed in HS^5 33 34^. In previous studies of HS in which single cell RNA-seq was performed on skin or mass cytometry by time of flight (CyTOF) was performed on blood, the cell types of interest included monocytes, B cells, plasma cells, dendritic cells (DCs), CD8 T cells, and CD4 T cells, which aligns with our PBMC CITEseq data^35 6 36^.

### 4.2 Overexpressed genes elucidate key cellular functions in HS

The upregulation of key inflammatory mediators such as *TNF, ZFP36, IL1B, JUN, NFKBIA*, and *MAP2K1* in CD14 monocytes and CD16 monocytes and cDC1/cDC2 signify their pivotal role in initiating and sustaining inflammation through the TNF pathway ^37^. The upregulation of genes like MKI67 and MMP9 indicates active monocyte participation in tissue remodeling and repair, essential processes in chronic inflammatory conditions like HS^38^. In healthy human skin, a skin-tropic population of TCM cells expressing skin-homing receptors *CCR7* and *L-selectin* has been observed, with overexpression in psoriasis and depletion by alemtuzumab therapy^39^. Consistent with these findings, our PBMC analysis also demonstrated increased expression of *CCR7* in CD8 TEM and TCM cells.

Elevated chemokine genes such as *CCL2/4* and *CXCL1/23/8* highlight the migration of immune cells to sites of inflammation^40 41^. Moreover, the enhanced expression of adhesion molecules (ITGB2, ICAM1,VCAM1, NCAM1, ICAM1) highlights the crucial role of monocyte and CD8 T cell interactions in cell trafficking to inflamed tissues^42^. The notable upregulation of NOD2 and CARD9 in monocytes highlight their potential role in responding to microbial antigens, further implicating these cells in the pathogenesis of HS^43 44^. Lastly, the metabolic reprogramming of monocytes, macrophages, or other immune cells as evidenced by the increased expression of (LDHA, GAPDH, PKM and HK2), reflects the heightened energy demands associated with sustaining an inflammatory response^44^

### 4.3 Pathway and cell-cell interaction analysis supports a pathogenic role of IL-17 and TNF signaling and plasmablasts in HS

HS has been strongly correlated with the Th17 immune response, with various studies finding elevated Th1 and Th17 associated cytokines (*IL-17, IFN-γ, IL-12, IL-23, IL-32, IL-1β*, and TNF) in HS tissue or serum as well as increased expression of genes associated with the *IL-17* signaling pathway (IFN family and JAK/STAT), suggesting a significant pathogenic role of *IL-17* ^13,14,45–48^. Indeed, anti-*IL17* targeted therapy (secukinumab) has shown significant clinical activity in HS, gaining FDA approval in 2023 ^49^. Here, utilizing pathway enrichment analysis to understand cell intrinsic mechanisms that contribute to HS we identified significant upregulation of the *IL-17* signaling pathway in HS, in agreement with prior studies. Interestingly, our analysis identified this upregulation in CD14 and CD16 monocyte. Previous studies have also found increased frequencies of CD14 and CD16 monocytes in HS, with aberrant expression of genes associated with *STAT1*/IFN-signaling ^50–52^. Interestingly, monocyte migration in rheumatoid arthritis (RA) has been shown to be mediated through *IL-17* signaling and p38 MAPK activation, with neutralization of *IL-17* resulting in suppression of monocyte migration into the RA synovial fluid ^53^. *IL-17* signaling has been shown to act through TNF receptor associated factors (TRAFs) as well as MAPK and *NF-κB* pathways ^54^. In keeping with these observations, we also observe upregulation of the TNF and MAPK signaling pathway in HS associated monocytes. Significantly, cell-cell interaction analysis also identified HS associated interactions between plasmablasts, expressing BTLA, and monocytes, expressing *TNFRSF14* (HVEM). Cell enrichment analysis also identifies both cell types to be significantly expanded in HS. Interestingly, plasmablasts were the most frequent partner in cell-cell interactions when considering interactions enriched in HS. Plasmablasts have been found to be highly elevated in HS skin lesions and shown to play a significant role in the pathogenesis of HS by contributing to a dysregulated immune response through the increased production of auto-antibodies ^55 56 6^. Additionally, tertiary lymphoid structures have been found in HS lesions that function similar to germinal centers, facilitating B-cell maturation and differentiation^55^. Interestingly, previous work has found that neutrophil and B-cell/plasma cell frequency are correlated and that neutrophils support B-cell activation and function by expressing BAFF, with B-cells expressing its receptors BAFFR, TACI, and BCMA^57^. While this interaction between neutrophil and B-cells is not observed in our data, likely due to cells being sourced from peripheral blood as opposed to the lesional skin, we do observe an interaction between BAFF-BAFFR and BAFF-TACI occurring between monocytes and plasmablasts, suggesting a similar mechanism of B-cell activation may be mediated by both neutrophils and monocytes in the pathogenesis of HS.

Overall, our findings highlight a potentially significant role of *IL-17* and TNF signaling in monocytes, as well as plasmablasts, in HS pathogenesis. Indeed, a previous study has shown that patients treated with anti-TNF therapy demonstrated a significant decrease in *IL-17*+ CD4 T-cells in lesional skin compared to treatment naïve patients ^40^. Taken together with our findings, this supports a potential synergistic role of anti-*IL17* and anti-TNF therapy in HS. Finally, plasmablasts, found to be elevated in HS skin lesions and, from our analysis, in peripheral blood, drives disease pathogenesis through the production of autoantibodies and is supported through interactions with BAFF, expressed on neutrophils^55 56 6 57^. Although our data does not show a similar direct interaction between neutrophils and B-cells, here we report a similar mechanism of B-cell activation in peripheral blood mediated by monocytes, expressing BAFFR and TCI. Together, these observations warrant further investigation into the precise cell-intrinsic and cell-extrinsic contributions of monocytes and plasmablasts to HS pathogenesis.

### 4.4 ML Classification of HS and Healthy Subjects based on single-cell RNA and protein markers

We observed that HS-associated differences in cell-specific gene and surface protein expression could distinguish HS from healthy subjects with > 0.95 AUROC achieved by several machine learning algorithms [Figure 5], though the general performance of these models may be limited by sample size (particularly for HS subjects). We nevertheless note that cell surface protein expression of CD325 (N-Cadherin) was consistently identified as an important marker in protein feature sets used for model training, which, given its biological significance as discussed above, may warrant further investigation as a diagnostic and therapeutic target. Besides modest cohort size, other limitations of this study include the sampling of patients from a single center and the use of only molecular biomarkers for subject classification. Future studies can address these limitations by recruiting a larger number of patients including those with other inflammatory skin diseases as well as by incorporating clinical and demographic data into the classification model.

### 4.5 Conclusion

This comprehensive analysis of differentially expressed genes and proteins across various immune cell types in the PBMC of HS provides valuable insights into the molecular underpinnings of this chronic inflammatory condition. The identified pathways and dysregulated genes and proteins offer potential targets for therapeutic interventions aimed at modulating the immune response in HS. The involvement of *Th2, Th17, Th23*, and *Th26*, TNF pathways in particular emphasize the need to explore targeted therapies that can address these specific immune dysregulations. Cell-cell interaction analysis supports a pro-inflammatory role for monocytes and B-cells mediated through TNF signaling and T-cell activation. For instance, we observed elevated expression of CD134/OX40, which has been targeted in clinical trials for HS and atopic dermatitis ^58 59^. Additionally, through machine learning, we identified elevated expression of several other cell surface marker [Figure 5 A–C] could serve as a new potential therapeutic and diagnostic marker in CD14 monocytes. Further research is warranted to elucidate the precise mechanisms and develop effective treatments for HS.

## Data Availability

The datasets generated for this study can be found in the GEO repository under the accession: GSE194315 for healthy and GSEXXX for HS samples
